# Long-term follow-up of a high- and a low-intensity smoking cessation intervention in a dental setting– a randomized trial

**DOI:** 10.1186/1471-2458-13-592

**Published:** 2013-06-19

**Authors:** Eva Nohlert, John Öhrvik, Åke Tegelberg, Per Tillgren, Ásgeir R Helgason

**Affiliations:** 1 Centre for Clinical Research, Uppsala University, Västerås, Sweden; 2 Department of Medicine, Karolinska Institutet, Stockholm, Sweden; 3 Faculty of Odontology, Malmö University, Malmö, Sweden; 4 School of Health, Care and Social Welfare, Mälardalen University, Västerås, Sweden; 5 Department of Public Health Sciences, Social Medicine, Karolinska Institutet and Centre for Epidemiology and Community Medicine, Stockholm, Sweden; 6 Reykjavik University, Reykjavik, Iceland

**Keywords:** Tobacco cessation, Treatment intensity, Public health, Health care, Questionnaire

## Abstract

**Background:**

Achieving lifelong tobacco abstinence is an important public health goal. Most studies use 1-year follow-ups, but little is known about how good these are as proxies for long-term and life-long abstinence. Also, intervention intensity is an important issue for development of efficient and cost-effective cessation treatment protocols.

The study aims were to assess the long-term effectiveness of a high- and a low-intensity treatment (HIT and LIT) for smoking cessation and to analyze to what extent 12-month abstinence predicted long-term abstinence.

**Methods:**

300 smokers attending dental or general health care were randomly assigned to HIT or LIT at the public dental clinic. Main outcome measures were self-reported point prevalence, continuous abstinence (≥6 months), and sustained abstinence. The study was a follow-up after 5–8 years of a previously performed 12-month follow-up, both by postal questionnaires.

**Results:**

Response rate was 85% (n=241) of those still alive and living in Sweden. Abstinence rates were 8% higher in both programs at the long-term than at the 12-month follow-up. The difference of 7% between HIT and LIT had not change, being 31% vs. 24% for point prevalence and 26% vs. 19% for 6-month continuous abstinence, respectively. Significantly more participants in HIT (12%) than in LIT (5%) had been sustained abstinent (p=0.03). Logistic regression analyses showed that abstinence at 12-month follow-up was a strong predictor for abstinence at long-term follow-up.

**Conclusions:**

Abstinence at 12-month follow-up is a good predictor for long-term abstinence. The difference in outcome between HIT and LIT for smoking cessation remains at least 5–8 years after the intervention.

**Trial registration number:**

NCT00670514

## Background

Tobacco use causes nearly six million deaths globally every year [1]. There is strong evidence that quitting smoking improves health and reduces the risk of premature death [2-4]. Achieving lifelong abstinence is therefore an important public health goal and thus a goal for tobacco control policies and smoking cessation treatment [5].

There is a vast literature on and strong evidence for the effectiveness of tobacco control programs [6], and a recent review concludes that interventions for tobacco cessation in the dental setting significantly can increase tobacco cessation rates [7]. It is also widely acknowledged that tobacco cessation programs are cost-effective, ranging from a few hundred to a few thousand dollars per Quality Adjusted Life Year (QALY) [6,8,9]. Behavioral support and medication, especially in combination, are effective and highly cost-effective as life-saving interventions [10]. The evidence for the efficacy of Nicotine Replacement Therapy (NRT) from clinical trials [6,11] has, however, in real-life situations, been somewhat conflicting [12-15]. There are a number of reported predictors for successful quitting, e.g. high motivation, low number of smoked cigarettes per day, high socioeconomic status, previous attempts (number and length), social support, gender (mostly being a man), and higher age [6,16,17].

The international literature indicates that 6-12% of smokers trying to quit are abstinent 12 months later if no support is available [6]. The majority of smokers make a number of quit attempts before they eventually achieve sustained abstinence [5,6]. Relapse to smoking after a quitting attempt is greatest in the first few weeks, then decreases rapidly over time and the longer the period of abstinence, the lower the rate of relapse [18-20]. Most studies report follow-up results up to 1 year [21], but less is known about to what extent being smoke-free at 12 months predicts more long-term abstinence. There is a general lack of clinical studies with long-term follow-up and, especially, studies that compare interventions with different intensity in a real-world clinical context not focusing on specific pharmaceuticals. Uncertainty remains regarding the optimal intensity of interventions, overall, and for different groups of smokers, but most studies indicate that high-intensity programs lead to higher rates of cessation [6,8,22-24]. The question of intensity of a smoking cessation program is important, because if a minimal intervention can result in even a small increase in cessation rates, this would have a large public health impact.

Even though Sweden has succeeded in reducing the prevalence of tobacco smoking, smokeless tobacco (snus) use is relatively common. Currently, 1.6 million Swedes (21% 2011) use tobacco every day, with great differences existing between different socioeconomic groups [25]. Although over 70% of daily smokers state that they want to quit (at least some time), the overall spontaneous smoking quit rate in the population has been only 1-2% per year [25]. Smoking is still the single most important risk factor for disease and premature death and, in coming decades, the supply of smoking cessation aids and support will be the most important elements of tobacco control [25,26]. However, the societal and, in particular, the healthcare system resources are strained to meet the need for cessation support, which emphasizes the need for further development of effective and cost-effective tobacco cessation methods [26,27].

The primary aim of the present study was to assess the long-term effectiveness of a high- and a low-intensity treatment for smoking cessation and the secondary aim was to analyze predictors for long-term abstinence. The basis for the present study is a randomized controlled trial (RCT) consisting of two programs of different intensity with a previous follow-up after 12 months [28].

## Methods

This study was a long-term follow-up of a previously reported study [28]. In the original intervention study, between August 2003 and February 2005, 300 smokers recruited via direct inquiry or advertising in dental or general health care were offered smoking cessation support performed in a dental setting. Inclusion criteria were daily smokers over 20 years of age. Excluded were individuals with reading difficulties and those not fluent in the Swedish language. The participants were randomly assigned to two study arms; one received high-intensity and the other low-intensity treatment support. The participants answered a baseline questionnaire and a follow-up questionnaire that was sent by mail 12 months after the planned smoking cessation date.

The long-term follow-up was performed 5–8 years after the planned smoking cessation dates. We developed a questionnaire with 13 questions, of which all were validated questions and eleven were previously used in the baseline and the 12-month questionnaires [28]. The questionnaire was pre-tested in 10 ex-smokers. The questions included smoking habits, amount, intention to quit (if still smoking), and use of snus, NRT, Champix®, Zyban®, and other support (social and/or other professional).

Before sending the questionnaires, the national population register was checked for deaths and emigration. The questionnaire was posted to the participants together with a cover letter and an informed consent form. Participants were asked to return the answered questionnaire and signed consent form in the enclosed prepaid envelope. Those still not answering after two written reminders were contacted and interviewed by telephone by a dental hygienist.

The original study, as well as the long-term follow-up, was approved by the ethical committee at Uppsala University (Dnr:Ups 02–457, Dnr: 2010/172).

### Treatment protocols

In the original study [28], all counselling was carried out by three dental hygienists educated and trained in general smoking cessation support methods who had received additional training in the specific programs used in the study.

The *High*-*Intensity Treatment* program (HIT) comprised eight 40-minute individual sessions over a period of four months. The program was a traditional state of the art smoking cessation program based on a mixture of behaviour therapy, coaching, and pharmacological advice.

The *Low*-*Intensity Treatment* program (LIT) consisted of one 30-minute counselling session focused on explaining the content of a traditional self-help program (in Swedish “Fimpa dig fri”). The leaflet contained an 8-week program with instructions and tasks to perform each week including several tests and behaviour registration exercises suggesting different action plans for different outcomes. In general, both the self-help and the clinic-based program were based on similar treatment protocols.

Information on possible benefits of NRT was included with both programs, but the participants received no recommendation regarding its use or not. At the first meeting, a smoking cessation date was fixed for all participants in both groups. The participants were informed that they would be followed-up. Both programs were free of charge.

### Questionnaire and outcome measures

*Abstinence* was assessed with the question: Have you smoked during the past seven days? The answer alternatives were: Yes, daily; Yes, but not daily; No, I have not smoked at all. Those who answered that they had not smoked during the past seven days answered an additional question about their last smoking date.

*Point prevalence abstinence* was defined as self-reported: “not one puff of smoke during the past seven days”, and *6*-*month continuous abstinence* as: “not one puff of smoke during the past six months (183 days)” [29]. Those reporting to be occasional smokers were classified as point prevalence abstinent, but as smokers according to 6-month continuous abstinence if they had not smoked during the past seven days. Those classified as *sustained abstinent* quit at the fixed smoking cessation date and had not smoked at all since then. (Additional file 1: Figure S1).

### Statistics

We used the “intention-to-treat” approach where all participants were included in the analyses according to the program they were randomized to. When measuring abstinence at follow-up, non-responders to the follow-up questionnaire were treated as smokers. However, we also analysed responder-only abstinence. For six individuals who did not reply to the baseline questionnaire, we only had information about their gender and which program they were randomized to, thus they could not be used in the analyses of background variables.

Chi-square test and Fisher´s exact test were used for comparisons between HIT and LIT for categorical variables and Mann–Whitney U-test for continuous variables. Wilcoxon Signed Rank Test was used to test change in number of smoked cigarettes at baseline and at long-term follow-up in still smokers. Logistic regression analysis was performed to calculate odds ratios (ORs) with a 95% confidence interval for the three different outcome measures. We first made univariable analyses of all relevant independent variables. As the purpose was to obtain a predictive model, statistical significant variables at p<0.2 in the univariable analyses at baseline and at 12-month follow-up, plus program and gender, were included in the multivariable analyses. The multivariable analyses were performed with forward and backward stepwise likelihood ratio test (LR), to detect potential collinearity that could disturb the analyses, with 5% for inclusion and 5% for exclusion. Hosmer and Lemeshow goodness of fit test was used for testing the overall fit of the logistic regression model [30]. The statistical analyses were performed using the Statistical Package for Social Sciences (SPSS, version 20.0). Statistical significance was set to p<0.05.

## Results

**Figure 1:**
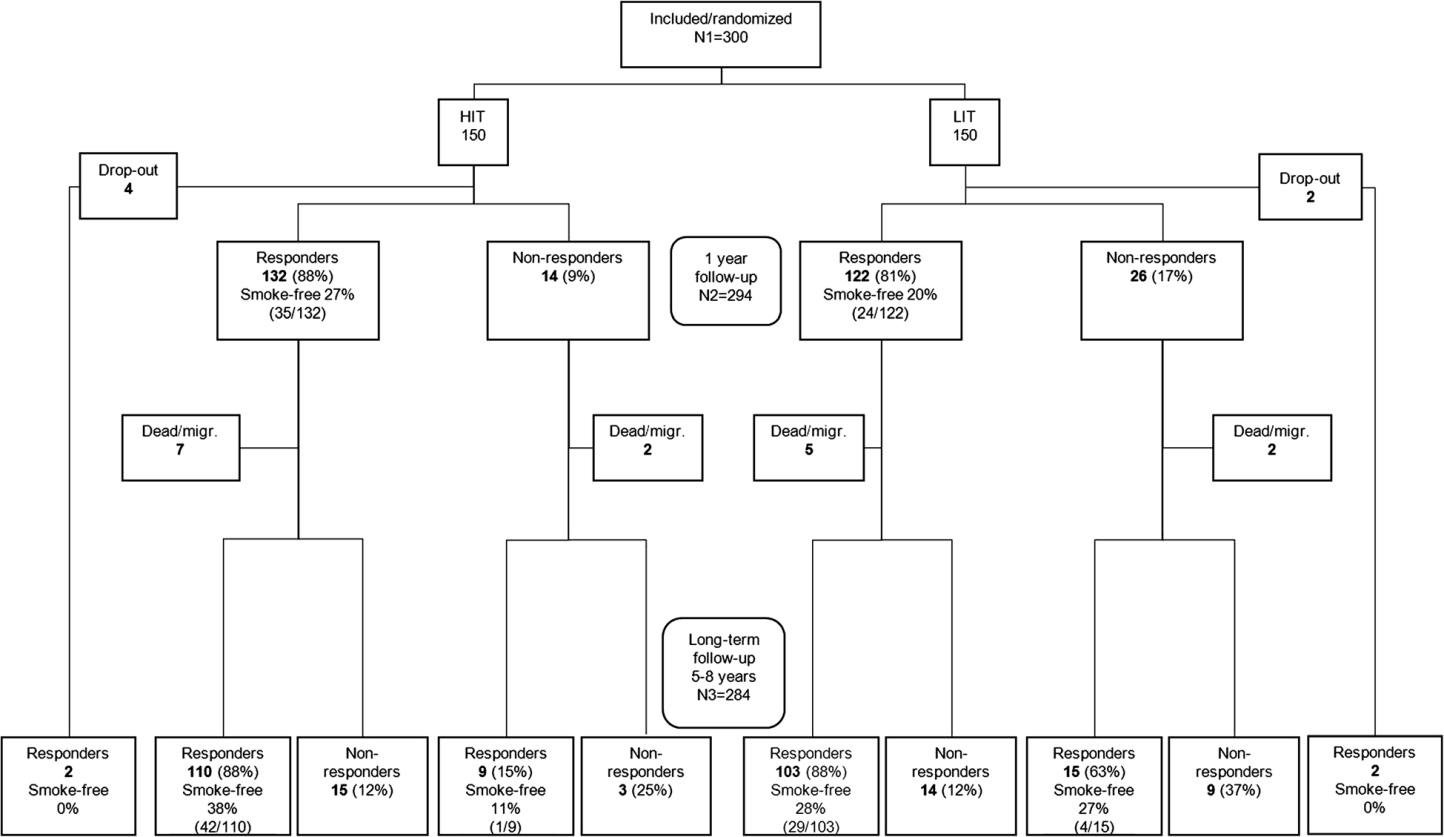
**Flowchart of the study.** (Through returned unanswered questionnaires with comments, information about smoking status at long-term follow-up was given for one in HIT (who was smokefree) and four in LIT (one was smoke-free). That is the reason for N_3_=284 while the sum of the lower boxes is 282).

Of the original 300 smokers, twelve had died and four had emigrated, leaving 284 possible individuals to contact for the long-term follow-up. Of these, 178 (63%) returned an answered questionnaire and thirteen returned an unanswered questionnaire but often with a comment which, in five cases, included information of their actual smoking status. By means of telephone calls to those 93 participants who had not returned the questionnaire, an additional 63 answers were retrieved, giving a total percentage of answers of 85% (241/284), 86% in HIT and 84% in LIT. Women constituted 81% of the respondents. The study´s flowchart is presented in Figure 1 and some baseline characteristics of the participants in Table 1.

**Table 1:** Population characteristics and abstinence at the 12-month and at the long-term follow-up, % (number) except for age

	**Total**	**High-intensity treatment**	**Low-intensity treatment**	p-value^*^
	**(N = 284)**			
		**(n**_**HIT **_**= 141)**	**(n**_**LIT **_**= 143)**	
**Gender:**				
Men	20 (58/284)	18 (26/141)	22 (32/143)	.410
Women	80 (226/284)	82 (115/141)	78 (111/143)	
**Age at baseline**^†^**:**				
mean (SD)	48.6 (10.3)	48.7 (9.6)	48.5 (11.0)	
median	49.0	48.0	49.0	.825
quartiles	42.0; 56.0	42.0; 56.0	41.0; 56.0	
**Education in years**^†^**:**				
0 - 9	22 (61/278)	18 (25/137)	26 (36/141)	.336
10 - 12	41 (115/278)	44 (60/137)	39 (55/141)	
≥ 13	37 (102/278)	38 (52/137)	35 (50/141)	
**Number of smoked cigarettes/week at baseline**^†^**:**				
mean (SD)	106 (45)	106 (50)	105 (40)	
median	105	105	105	.794
quartiles	70; 140	70; 140	70; 140	
**Intention to quit at baseline**^†^**:**				
not within 6 months	1 (4/278)	1 (1/137)	2 (3/141)	.120^¶^
within 6 months	50 (139/278)	46 (63/137)	54 (76/141)	
within 1 month	48 (133/278)	52 (71/137)	44 (62/141)	
trying just now	1 (2/278)	1 (2/137)	0 (0/141)	
**12-month follow-up:**				
Point prevalence abstinence^‡^				
of which:	19 (55/284)	23 (32/141)	16 (23/143)	.159
< 6 months	5 (15/284)	4 (6/141)	6 (9/143)	.443
≥ 6 months^§^	14 (40/284)	18 (26/141)	10 (14/143)	.036
**Long-term follow-up:**				
Point prevalence abstinence	27 (78/284)^‡^	31 (44/141)^‡^	24 (34/143)^‡^	.161
	31 (76/241)^║^	35 (43/121)^║^	27 (33/120)^║^	.179
6-month continuous abstinence	22 (63/284)^‡^	26 (36/141)^‡^	19 (27/143)^‡^	.177
	25 (61/241)^║^	29 (35/121)^║^	22 (26/120)^║^	.195
Sustained abstinence	9 (24/284)^‡^	12 (17/141)^‡^	5 (7/143)^‡^	.030
	10 (24/241)^║^	14 (17/121)^║^	6 (7/120)^║^	.033

^*^ Statistical significant difference between HIT and LIT tested with chi-square test, except for age and number of cigarettes at baseline which were tested with Mann–Whitney U-test.

^†^ Information from 278 participants (137 HIT, 141 LIT) because six did not reply to the baseline questionnaire.

^‡^ According to intention-to-treat, non-responders treated as smokers.

^§^ Equal to 6-month continuous abstinence.

^║^ Responder-only abstinence.

^¶^ p-value for grouping into “not within 6 months/within 6 months” vs. “within 1 month/trying just now” because 4 cells (50.0%) had expected count less than 5 in the original grouping.

The median follow-up time was 6.2 (q_1_ 5.9; q_3_ 6.7) years (HIT median 6.1, q_1_ 5.8; q_3_ 6.7, LIT median 6.3, q_1_ 6.0; q_3_ 6.8, p=0.135). It was measured from the planned cessation date until the date when the questionnaire was completed or the telephone interview was performed.

### Abstinence/outcome, length of abstinence/time to first stop

**Figure 2:**
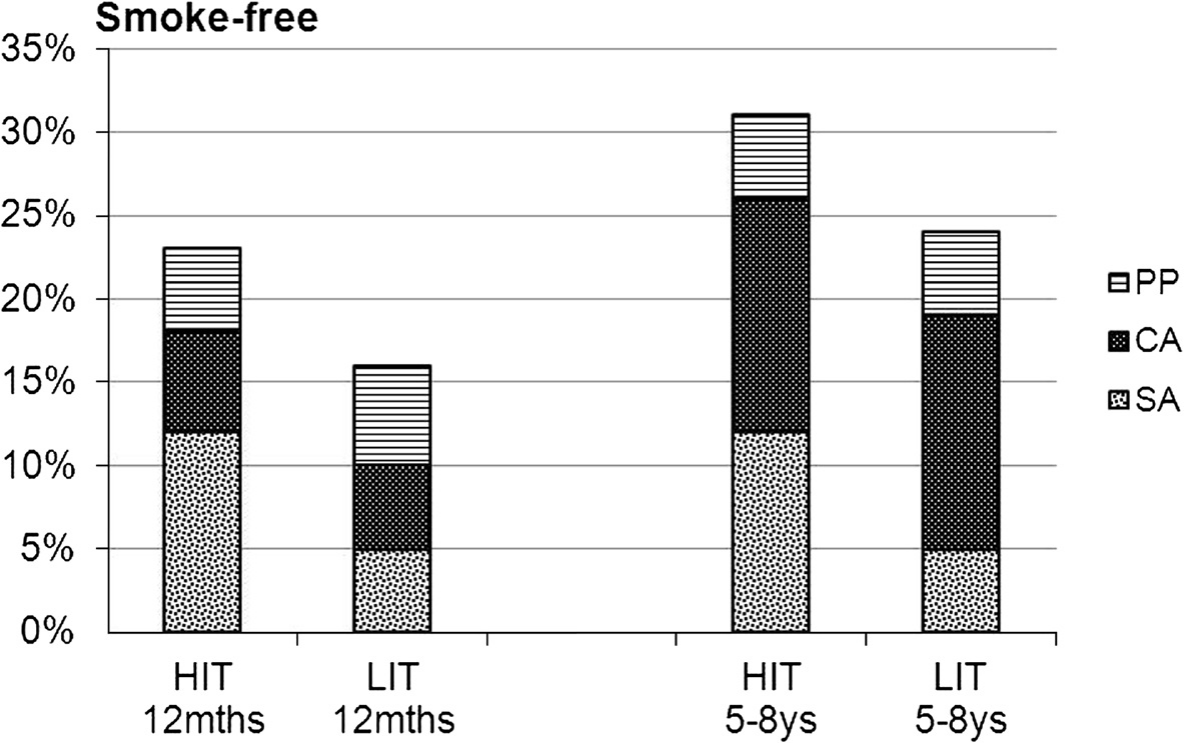
**Outcomes for HIT and LIT at 12-month and at long-term (5–8 ys) follow-up.** (PP=point prevalence abstinence, CA=6-month continuous abstinence, SA = sustained abstinence).

Point prevalence was 31% in HIT and 24% in LIT, and 6-month continuous abstinence 26% in HIT and 19% in LIT. These differences in abstinence rates between the programs were not statistically significant. However, when analyzing the proportion of participants who had maintained sustained abstinence, there was a significant difference in favor of HIT: 12% vs. 5% (p=0.030). The differences in quit rates between HIT and LIT and the p-values were almost the same in ITT- and in responder-only analyses (Table 1, Figure 2).

The mean number of smoke-free days immediately before the long-term follow up was 1317 (SD 942) in HIT and 1194 (SD 894) in LIT (not significant). However, the number of days from baseline assessment to first quit was significantly shorter in HIT (median 185) than in LIT (median 502) (p=0.025).

### Amount of cigarettes

The number of smoked cigarettes per week decreased from baseline (median=105, interquartile range=70) to long-term follow-up (median=84, interquartile range=49) in still smokers with median=14, interquartile range=40; p<0.001. This reduction was significantly higher in HIT than in LIT (p=0.042).

### Other support

About two-thirds of the participants stated that they had had access to other support, mainly social (from family, friends, and workmates). Abstinence rates were significantly higher among those with other support than among those without, regarding point prevalence 36% vs. 21% (p=0.017) and 6-month continuous abstinence 29% vs. 16% (p=0.034). However, 10% had maintained sustained abstinence, both among those with and without other support.

Access to other support was equal in both programs, but in LIT there were significantly higher abstinence rates among those with other support than among those without. Point prevalence was 33% vs. 12% (p=0.025) and 6-month continuous abstinence 27% vs. 9% (p=0.040).

### Gender differences

Women had higher, although non-significant, abstinence rates than men at the long-term follow-up. Point prevalence was 30% among women and 17% among men (p=0.051), and 6-month continuous abstinence 24% among women and 15% among men (p=0.171). The difference between women and men was larger at the long-term than at the 12-month follow-up. Access to other support was significantly higher among women (73%) than among men (53%) (p=0.015).

When analyzing gender differences in HIT and LIT separately, the only significant difference was that, in HIT, more women than men had access to other support (p=0.003).

### Pharmaceuticals

Twenty-two percent of all participants had used pharmaceuticals for smoking cessation (NRT, Zyban®, Champix®) during the week before the long-term follow-up, compared with 16% at the 12-month assessment (NRT) and 8% at baseline (NRT), with similar proportions in both programs.

Since the program-start, 24% had used NRT ≥5 weeks, 30% <5 weeks, and 47% had not used it at all, with similar proportions in HIT and LIT. The effect of NRT-use on abstinence did not differ significantly between HIT and LIT (Additional file 2: Table S1). No difference in abstinence rates was detected between those who had used NRT ≥5 weeks and those who had not used NRT at all since the program start. However, those who had used NRT <5 weeks were significantly less likely to be abstinent compared with those who had used it ≥5 weeks or not at all. We found a significant association between NRT-use (none or ≥5 weeks compared to <5 weeks) and abstinence, controlling for program (for point prevalence OR=0.31, p=0.001 and for 6-month continuous abstinence OR=0.40, p=0.017) (Additional file 3: Table S2). Zyban® was used by 14% (31/224) and Champix® by 14% (31/224) since the program-start, with similar proportions in both programs.

### Snus

Eight percent used snus during the week before the long-term follow-up, compared with 6% at the 12-month assessment and 7% at baseline. There were slightly higher proportions in HIT than in LIT at all assessments, but not statistically significant.

### Still trying

Of the participants responding to the follow-up and who were still smoking, 63% (105/166) were actually trying to quit at that time or intended to make a new attempt within the following six months, with no significant differences between the programs.

### Logistic regression analyses

In the multivariable analyses, we adjusted for significant confounders up to the 12-month follow-up, plus program and gender and the results are presented in Table 2. Point prevalence and 6-month continuous abstinence at the 12-month follow-up were strong predictors for point prevalence as well as for 6-month continuous abstinence at the long-term follow-up, when controlled for program and gender. Program (HIT) was the only significant predictor of sustained abstinence, controlled for gender and NRT use between baseline and 12-month follow-up. The results of the univariable analyses for the three outcomes, respectively, are presented in Additional file 4: Table S3, Additional file 5: Table S4 and Additional file 6: Table S5.

**Table 2:** Multivariable logistic regression analyses^* ^**for point prevalence abstinence**^**†**^**, 6-month continuous abstinence**^**†**^**, and sustained abstinence**

**Point prevalence abstinence **^**†**^			
**Variable**	**OR**	**95% CI for OR**	**p-value**
^‡^ Program; HIT vs. LIT (ref)	1.21	0.59-2.49	.599
^‡^ Gender; men vs. women (ref)	0.54	0.20-1.46	.226
^‡^ Point prevalence at 12-month follow-up; yes vs. no (ref)	13.82	6.44–29.65	<.001
^‡^ Other support at 12-month follow-up; yes vs. no (ref)	4.37	1.13–16.95	.033
^§^ Program; HIT vs. LIT (ref)	1.12	0.56-2.26	.751
^§^ Gender; men vs. women (ref)	0.67	0.26-1.71	.405
^§^ 6-month continuous abstinence at 12-month follow-up; yes vs. no (ref)	13.53	5.66–32.32	<.001
^§^ Other support at 12-month follow-up; yes vs. no (ref)	4.95	1.30–18.85	.019
**6-month continuous abstinence **^**†**^			
**Variable**	**OR**	**95% CI for OR**	**p-value**
^║^ Program; HIT vs. LIT (ref)	1.26	0.65-2.43	.487
^║^ Gender; men vs. women (ref)	0.55	0.23-1.35	.193
^║^ Point prevalence at 12-month follow-up; yes vs. no (ref)	14.10	7.05–28.19	<.001
^¶^ Program; HIT vs. LIT (ref)	1.10	0.57-2.13	.779
^¶^ Gender; men vs. women (ref)	0.62	0.26-1.51	.297
^¶^ 6-month continuous abstinence at 12-month follow-up; yes vs. no (ref)	18.70	8.30–42.16	<.001
Sustained abstinence **			
**Variable**	**OR**	**95% CI for OR**	**p-value**
Program; HIT vs. LIT (ref)	3.44	1.34–8.83	.010
Gender; men vs. women (ref)	1.21	0.41-3.60	.733

^*^ Performed with variables significant at p<0.02 in the univariable analyses, only including variables up to 12-month follow-up.

^**†**^ Separate models for point prevalence at 12-month follow-up and for 6-month continuous abstinence at 12-month follow-up.

^**‡**^ N=210, Nagelkerke R-Square 39.2%, Hosmer and Lemeshow test of goodness of fit p=0.884.

^§^ N=210, Nagelkerke R-Square 33.5%, Hosmer and Lemeshow test of goodness of fit p=0.920.

^║^N=284, Nagelkerke R-Square 31.7%, Hosmer and Lemeshow test of goodness of fit p=0.969.

^¶^ N=284, Nagelkerke R-Square 30.7%, Hosmer and Lemeshow test of goodness of fit p=0.932.

** N=210, Nagelkerke R-Square 11.7%, Hosmer and Lemeshow test of goodness of fit p=0.970.

### Transitions between smoking statuses from 12-month to long-term follow-up

At 12-month follow-up the participants were in one of three smoking statuses: smokers, point prevalent but <6 months abstinent or 6-month continuous abstinent. Here we describe the transition between smoking statuses from 12-month to long-term follow-up for these three statuses. Among those who were *smokers* at the 12-month follow-up, 83% were still smokers at the long-term follow-up and 17% were smokefree (12% had been abstinent for at least 6 months), with equal proportions in HIT and LIT.Among those who were *point prevalent but* <*6 months* at the 12-month follow-up, 40% were smokers at the long-term follow-up and 60% were smokefree (40% had been abstinent for at least 6 months). In this group the program effects differed: in HIT 17% were smokers and 83% were smoke-free (50% had been abstinent for at least 6 months), and in LIT 56% were smokers and 44% were smoke-free (33% had been abstinent for at least 6 months). However, since there were only 15 participants in this group, it was not possible to test statistical significance between the programs. Among those who were *6*-*month continuous abstinent* at the 12-month follow-up, 23% were smokers at the long-term follow-up and 77% were smokefree (75% had been abstinent for at least 6 months). In this group the difference between HIT and LIT was smaller; however, a larger proportion smoked at the long-term follow-up in LIT than in HIT, and a larger proportion was smokefree in HIT than in LIT. (Additional file 7: Figure S2).

In *drop*-*out analyses* we compared the baseline characteristics and 12-month variables of responders (n=241) with non-responders (n=43) at the long-term follow-up. The non-responders had higher cigarette consumption at baseline and at 12-month follow-up (p=0.010 and p=0.025). No other differences were seen between the responders and non-responders. We also compared those answering via questionnaire with those answering via telephone, and found more non-smokers among the questionnaire-responders and more smokers among the telephone-responders (p=0.001). Five participants who did not reply to the 12-month follow-up were smoke-free at the long-term follow-up.

## Discussion

At the long-term follow-up 5–8 years after the planned smoking cessation date, 27% of the participants were abstinent. Point prevalence was 31% in HIT and 24% in LIT, of which 26% and 19%, respectively, was 6-month continuous abstinence. Sustained abstinence was maintained by 12% in HIT and 7% in LIT (p=0.03). The 7% difference in abstinence rates between HIT and LIT remained unchanged over time and may be considered clinically significant because of the very large health gains that accrue from stopping smoking and which are attached to cost-savings [31]. The proportion of smoke-free participants had increased by 8% from the 12-month to the long-term follow-up in both treatment arms. Abstinence at the 12-month follow-up was a strong predictor for abstinence at the long-term follow-up. A probable theoretical explanation of the observed difference between the treatment arms is that structured long-term contact may increase the possibilities for being exposed to positive reinforcement and skills training delivered by the counsellor.

Returning to our question in the introduction: how good are 1-year follow-ups as proxies for long-term and life-long abstinence? Other studies report that, among 12-month abstainers, 60-70% remains abstinent for at least eight years [21,32]. We found that abstinence rates increased by 8% from 12-month to long-term (5-8 yrs) follow-up in both programs, and that abstinence at 12-month follow-up was the strongest predictor of long-term abstinence. However, regarding differences between the programs, we found that the group with point prevalence but <6 months at the 12-month follow-up was crucial; HIT had more abstinent participants and fewer smokers at long-term follow-up compared with LIT. Thus, a possibility could be to offer some kind of renewed support after one year to participants in this group, which needs to be studied further.

As far as we know, there is a lack of non-pharmacological clinical studies with long-term follow-up and, consequently, it is difficult to compare abstinence rates. A cessation program in a specialist clinic in Sweden, with an average of 10 treatment sessions and NRT as a recommended part of the program, shows 40% abstinence after 5–7 years, of which 18% is continuous abstinence [33]. In the comprehensive American Lung Health Study (LHS) with long-term follow-up, smokers with mild airway obstruction are randomized to either an intensive 12-week smoking cessation intervention (SI) including nicotine gum and repeated follow-up visits/contacts or to usual care (UC). Point prevalence abstinence is high and increases in both programs over time, in SI 39% after 5 years and 52% after 11 years, in UC 22% and 43%, respectively. Participants with sustained abstinence for the first 5-year period are very likely to still be abstinent after 11 years, when validated sustained abstinence is 22% for SI and 6% for UC [34,35]. Blondal et al. report 9% sustained abstinence after six years among smokers in Iceland receiving nicotine patches for five months and 10 individual and group meetings [36].

In Sweden, 85-90% of the adult inhabitants regularly visit the dental care [25,37]. The present study, performed in a public dental setting, supports the findings of benefits by using dental professionals for cessation interventions presented in the recent Cochrane-review [7]. The review finds a significant effect of behavioral interventions versus control for adult smokers in general dental practices with 6–12 months of follow-up (OR 2.38, 95% CI 1.70-3.35, no evidence of heterogeneity [I^2^=3%]). Consequently, the dental setting is a potential public health arena.

The majority of smokers quit and relapse a number of times before they achieve sustained abstinence [5,6]. The estimated annual incidence of relapse to smoking after one year of abstinence is 2-15% in retrospective studies (with recall problems) and about 10% in prospective studies [19]. Zhu et al. show that multiple compared with single counselling and, especially a self-help intervention, significantly lower the relapse rate during the first week, which in turn translates into a higher 12-month abstinence rate [38]. This is in accordance with our results that significantly more participants in HIT than in LIT maintained sustained abstinence. We were not able to comment on how the participants in the present study possibly quit and relapsed, since we decided not to use the *quitting attempts* information. The reasons were the long follow-up period, adherent recall bias problems [39], a considerable internal drop-outs on the question of quitting attempts, and the study design with no measurements between baseline, 12-month and long-term follow-ups.

In the present study, abstinence at the 12-month follow-up was a very strong predictor of abstinence at the long-term follow-up, with ORs between 13.53 and 18.70 in the multivariable logistic regression analyses. Program was the only significant predictor of maintaining sustained abstinence, with an OR of 2.67 for HIT vs. LIT. Among common reported predictors for successful quitting [6,16,17], only *other support* at the 12-month follow-up was a significant predictor for point prevalence at the long-term follow-up in the present study (Table 2).

Women had higher, however not statistically significant, quitting rates than men in the present study. One explanation can be that *gender* interacts with *other support*, as women were significantly more likely than men to have access to it. These gender differences in access to social support correspond to population-based data from Sweden assessing availability of emotional support [40].

We did not find any strong support for the positive long-term effect of *NRT* use in the present study. No difference was detected in abstinence rates between those who had used NRT ≥5 weeks and those who had not used NRT at all, while those who had used NRT <5 weeks were significantly less likely to be abstinent (Additional file 2: Table S1, Additional file 3: Table S2). However, he study was not designed to assess NRT use as such, so we are not able to draw conclusions regarding the real-life effect of NRT. Abstinence rates at 12 months are significantly improved by NRT use in self-quitters without formal behavioral support [15], as well as among those using NRT ≥5 weeks at the Swedish National Tobacco Quitline [14]. We did not detect such an effect at the 12-month follow-up in our original study [28]. The contrasts concerning effectiveness of NRT in clinical trials [6,11,21] and population studies [12,41] need further investigation not least in the light of policy and economic coverage decisions.

The role of *snus* as a substitute for smoking was low (≤8%) in the present study, and was not a significant predictor for abstinence in the multivariable analyses. Snus seems to play an insignificant role for cessation in smokers with professional support [14,42], however, presumably plays a greater role in self-quitters [43].

The still smokers had reduced their *number of smoked cigarettes* significantly, which can be seen as a step in the process towards quitting [29,44]. And two-thirds of this group intended to make a new quit attempt within the following six months. It is interesting what happened with *intention to quit* from 12-month to long-term follow-up. Among smokers at the 12-month follow-up who intended to make a new quit attempt within the following six months, 18% were smoke-free at the long-term follow-up. However, among those who did not intend to quit at the 12-month follow-up, 36% were smoke-free at the long-term follow-up (p=0.001).

The *generalizability* of a clinical trial is an important issue. For the present study we approached adult daily smokers fluent in the Swedish language and invited them to participate. Those interested in smoking cessation were then randomized [28]. This means that they were more motivated to quit than smokers in the general population, but less motivated than, for example, smokers who call a quitline [14,45], and who have themselves taken contact with a smoking cessation expert. Smoking ≤10 cigarettes/day is a criterion reported to lead to exclusion for a large percentage of individuals in clinical trials [46], but this was not applied in the present study since it would have reduced the generalizability.

Strengths of the present study are: i) the randomized controlled trial-design, ii) the long-term follow-up period, and iii) the high response rate. Limitations are: i) the lack of chemical validation of abstinence and ii) the risk of memory bias. However, self-reports are considered accurate in most smoking cessation studies [47] and as participants were free to use snus and NRT, it would be problematic to distinguish low levels of smoking and use of snus or NRT. Furthermore, we had no reason to assume a different distribution of untruthful answers in the two arms. The risk of memory bias increases with length of follow-up and we noted discrepancies between baseline, 12-month, and long-term follow-up questionnaires regarding information of last date of smoking and number of smoke-free days for 22 participants in HIT and 17 in LIT (16% of all responders).

## Conclusion

Abstinence at 12-month follow-up is a good predictor for long-term abstinence. The difference in outcome between HIT and LIT for smoking cessation, although non-significant except for sustained abstinence, remained relatively constant and in favour of HIT for at least 5–8 years after the intervention.

### Implications

A 12-month follow-up may be a sufficient end-point for assessment of smoking cessation programs with fixed quit dates. Also, which the present and the prior study suggests [9,28], a relatively intensive program may be more effective and cost-effective than a less intensive program.

## Competing interests

The authors declare that they have no competing interests.

## Authors´ contributions

EN: construction and validation of outcome measures, data collection and analysis, manuscript preparation, JÖ: statistical consultation, manuscript preparation, ÅT: study design, manuscript preparation, PT: study design, manuscript preparation, ARH: study design, construction and validation of outcome measures, data analysis, manuscript preparation. All authors read and approved the final manuscript.

## Pre-publication history

The pre-publication history for this paper can be accessed here:

http://www.biomedcentral.com/1471-2458/13/592/prepub

## Supplementary Material

Additional file 1: Figure S1 Classification of predictor and outcome variables used in the present study. Click here for file

Additional file 2: Table S1 Outcome at long-term follow-up in HIT and LIT according to NRT use between baseline and long-term follow-up. Click here for file

Additional file 3: Table S2 Outcome at long-term follow-up according to NRT use controlled for program (logistic regression analysis). Click here for file

Additional file 4: Table S3 Univariable logistic regression analyses for point prevalence abstinence at long-term follow-up. Click here for file

Additional file 5: Table S4 Univariable logistic regression analyses for 6-month continuous abstinence at long-term follow-up. Click here for file

Additional file 6: Table S5 Univariable logistic regression analyses for sustained abstinence. Click here for file

Additional file 7: Figure S2 Transitions between smoking statuses from 12-month to long-term (5-8ys) follow-up. Click here for file
