# Supplementary material for: Long-term follow-up of a high- and a low-intensity smoking cessation intervention in a dental setting– a randomized trial
Source: BMC Public Health. 2013 Jun 19;13:592. doi: 10.1186/1471-2458-13-592 (PMC3693879; doi:10.1186/1471-2458-13-592)
Supplement: Additional file 1: Figure S1 — Classification of predictor and outcome variables used in the present study. [file 1471-2458-13-592-S1.pdf]

**Smoke-free period**	**Predictors at 12-month follow-up**	**Outcome variables at long-term follow-up**		
< past week	Smoker	Smoker		
≥ past week	Point prevalence			
≥ past 6 months	6-month continuous abstinence			Point prevalence abstinence
Smoke-free ever since the program		Sustained abstinence	6-month continuous abstinence	
