# Supplementary material for: Long-term follow-up of a high- and a low-intensity smoking cessation intervention in a dental setting– a randomized trial
Source: BMC Public Health. 2013 Jun 19;13:592. doi: 10.1186/1471-2458-13-592 (PMC3693879; doi:10.1186/1471-2458-13-592)
Supplement: Additional file 2: Table S1 — Outcome at long-term follow-up in HIT and LIT according to NRT use between baseline and long-term follow-up. [file 1471-2458-13-592-S2.doc]

Additional Table 1. Outcome at long-term follow-up in HIT and LIT according to NRT use between baseline and long-term follow-up

| **%** (n/N) |  |  | **Total** | **HIT** | **LIT** | **p-value*** |
| --- | --- | --- | --- | --- | --- | --- |
| **NRT use between baseline and long-term follow-up** | None | PP 5-8ys | 38 (40/105) | 41 (22/54) | 35 (18/51) | .483 |
|  | <5 weeks | PP 5-8ys | 16 (11/67) | 23 (7/30) | 11 (4/37) |  |
|  | ≥5 weeks | PP 5-8ys | 41 (22/53) | 40 (12/30) | 43 (10/23) |  |
|  |  |  |  |  |  |  |
|  | None | CA 5-8ys | 31 (32/105) | 35 (19/54) | 25 (13/51) | .530 |
|  | <5 weeks | CA 5-8ys | 15 (10/67) | 20 (6/30) | 11 (4/37) |  |
|  | ≥5 weeks | CA 5-8ys | 32 (17/53) | 30 (9/30) | 35 (8/23) |  |

PP 5-8ys = point prevalence abstinence at long-term follow-up (5-8ys), CA 5-8ys = 6-month continuous abstinence at long-term follow-up (5-8ys) ***** Test of interaction between program and NRT use, i.e. if the effect of NRT on abstinence differs between HIT and LIT.
