# Supplementary material for: Long-term follow-up of a high- and a low-intensity smoking cessation intervention in a dental setting– a randomized trial
Source: BMC Public Health. 2013 Jun 19;13:592. doi: 10.1186/1471-2458-13-592 (PMC3693879; doi:10.1186/1471-2458-13-592)
Supplement: Additional file 3: Table S2 — Outcome at long-term follow-up according to NRT use controlled for program (logistic regression analysis). [file 1471-2458-13-592-S3.doc]

Additional Table 2. Outcome at long-term follow-up according to NRT use controlled for program (logistic regression analysis)

|  | **Point prevalence abstinence** (PP) | | | **6-month continuous abstinence** (CA) | | |
| --- | --- | --- | --- | --- | --- | --- |
| **Variable** | **n/N** | **OR (95% CI for OR)** | **p-value** | **n/N** | **OR (95% CI for OR)** | **p-value** |
| NRT*****use between baseline and  long-term follow-up; |  |  |  |  |  |  |
| - none or ≥ 5 weeks (ref) | 158/225 | 1.0 |  | 158/225 | 1.0 |  |
| - < 5 weeks | 67/225 | 0.31 (0.15-0.64) | .001 | 67/225 | 0.40 (0.19-0.85) | .017 |
| Program; |  |  |  |  |  |  |
| - LIT (ref) | 111/225 | 1.0 |  | 111/225 | 1.0 |  |
| - HIT | 114/225 | 1.31 (0.73-2.32) | .362 | 114/225 | 1.39 (0.76-2.56) | .285 |

***** Max number of weeks for any preparation

Nagelkerke R-Square PP 7.8% , CA 5.1%
