# Supplementary material for: Long-term follow-up of a high- and a low-intensity smoking cessation intervention in a dental setting– a randomized trial
Source: BMC Public Health. 2013 Jun 19;13:592. doi: 10.1186/1471-2458-13-592 (PMC3693879; doi:10.1186/1471-2458-13-592)
Supplement: Additional file 4: Table S3 — Univariable logistic regression analyses for point prevalence abstinence at long-term follow-up. [file 1471-2458-13-592-S4.doc]

Additional Table 3. Univariable logistic regression analyses for point prevalence abstinence at long-term follow-up

| **Variable** | **n/N*** | **OR (95% CI for OR)** | **p-value** |
| --- | --- | --- | --- |
| Program; HIT vs. LIT (ref) | 141/284 vs. 143/284 | 1.45 (0.86-2.46) | .162 |
| Gender; men vs. women (ref) | 58/284 vs. 226/284 | 0.48 (0.23-1.01) | .054 |
| Age at baseline | md=49, Q_1_=42, Q_3_=56, N=278 | 1.00 (0.97-1.02) | .831 |
| Education; - 0-9 years (ref) - 10-12 years - ≥ 13 years | 61/278 115/278 102/278 | 1.0 0.99 (0.49-2.01) 1.29 (0.63-2.61) | .984 .486 |
| Number of years smoked before baseline | md=30, Q_1_=21, Q_3_=35, N=276 | 0.99 (0.97-1.02) | .451 |
| Number of cigarettes at baseline | md=105, Q_1_=70, Q_3_=140, N=278 | 0.99 (0.99-1.00) | .054 |
| Smokefree ≥1 week sometime before baseline; yes vs. no (ref) | 217/278 vs. 61/278 | 2.33 (1.12-4.86) | .024 |
| Smokefree ≥1 week sometime before baseline, number of times;  ≥5 vs. 0-4 (ref) | 63/278 vs. 215/278 | 1.52 (0.84-2.78) | .170 |
| Max length of earlier smoke-free period, number of months | md=3, Q_1_=1, Q_3_=12, N=216 | 1.01 (1.00-1.02) | .223 |
| Stages-of-change at baseline; preparation/action vs. precontemplation/contemplation (ref) | 135/278 vs. 143/278 | 1.34 (0.79-2.27) | .272 |
| Snus use the week before baseline; yes vs. no (ref) | 19/278 vs. 259/278 | 2.48 (0.97-6.36) | .059 |
| NRT use the week before baseline; yes vs. no (ref) | 22/278 vs. 256/278 | 0.38 (0.11-1.33) | .129 |
| Other support at baseline; yes vs. no (ref) | 266/278 vs. 12/278 | 2.00 (0.43-9.34) | .378 |
| Passive smoking at baseline; not exposed vs. exposed (ref) | 182/278 vs. 96/278 | 1.17 (0.67-2.04) | .587 |
| Smoking-status at 12-month follow-up; - smoker (ref) - point prevalence but <6 months - 6-month continuous abstinence | 229/284 15/284 40/284 | 1.0 7.54 (2.54-22.43) 17.31 (7.63-39.30) | <.001 <.001 |
| Snus use the week before 12-month follow-up; yes vs. no (ref) | 12/209 vs. 197/209 | 1.71 (0.52-5.62) | .375 |
| NRT use the week before 12-month follow-up; yes vs. no (ref) | 34/210 vs. 176/210 | 0.79 (0.35-1.81) | .580 |
| Other support at 12-month follow-up; yes vs. no (ref) | 176/210 vs. 34/210 | 5.48 (1.61-18.66) | .006 |
| Passive smoking at 12-month follow-up; not exposed vs. exposed (ref) | 153/207 vs. 54/207 | 0.86 (0.44-1.67) | .655 |
| Compliance at 12-month follow-up; high vs. medium, low or no (ref) | 60/210 vs. 150/210 | 1.65 (0.88-3.11) | .119 |
| Snus use the week before long-term follow up; yes vs. no (ref) | 17/218 vs. 201/218 | 3.44 (1.25-9.46) | .017 |
| Drug^†^ use the week before long-term follow-up; yes vs. no (ref) | 52/238 vs. 186/238 | 0.91 (0.47-1.77) | .782 |
| NRT**^‡^** use between baseline and long-term follow-up; - none (ref) - < 5 weeks - ≥ 5 weeks | 105/225 67/225 53/225 | 1.0 0.32 (0.15-0.68) 1.15 (0.59-2.26) | .003 .678 |
| Zyban® use between baseline and long-term follow-up; - none (ref) - < 7 weeks - ≥ 7 weeks | 194/225 21/225 10/225 | 1.0 0.20 (0.05-0.88) 1.26 (0.35-4.63) | .034 .724 |
| Champix® use between baseline and long-term follow-up; - none (ref) - < 12 weeks - ≥ 12 weeks | 195/225 22/225 8/225 | 1.0 0.44 (0.14-1.37) 2.00 (0.49-8.25) | .157 .338 |
| Other support at long-term follow-up; yes vs. no (ref) | 163/236 vs. 73/236 | 2.19 (1.14-4.21) | .018 |

^*^n=number in category, N=total number in analysis ^†^Including NRT, Zyban®, and Champix®.
**^‡^**Max number of weeks for any preparation.
