# Supplementary material for: Long-term follow-up of a high- and a low-intensity smoking cessation intervention in a dental setting– a randomized trial
Source: BMC Public Health. 2013 Jun 19;13:592. doi: 10.1186/1471-2458-13-592 (PMC3693879; doi:10.1186/1471-2458-13-592)
Supplement: Additional file 5: Table S4 — Univariable logistic regression analyses for 6-month continuous abstinence at long-term follow-up. [file 1471-2458-13-592-S5.doc]

Additional Table 4. Univariable logistic regression analyses for 6-month continuous abstinence at long-term follow-up

| **Variable** | **n/N*** | **OR (95% CI for OR)** | **p-value** |
| --- | --- | --- | --- |
| Program; HIT vs. LIT (ref) | 141/284 vs. 143/284 | 1.47 (0.84-2.59) | .179 |
| Gender; men vs. women (ref) | 58/284 vs. 226/284 | 0.59 (0.27-1.27) | .174 |
| Age at baseline | md=49, Q_1_=42, Q_3_=56, N=278 | 1.01 (0.98-1.03) | .644 |
| Education; - 0-9 years (ref) - 10-12 years - ≥ 13 years | 61/278 115/278 102/278 | 1.0 1.13 (0.53-2.45) 1.40 (0.65-3.03) | .749 .396 |
| Number of years smoked before baseline | md=30, Q_1_=21, Q_3_=35, N=276 | 1.00 (0.97-1.02) | .779 |
| Number of cigarettes at baseline | md=105, Q_1_=70, Q_3_=140, N=278 | 0.99 (0.99-1.00) | .037 |
| Smokefree ≥1 week sometime before baseline; yes vs. no (ref) | 217/278 vs. 61/278 | 2.68 (1.15-6.24) | .022 |
| Smokefree ≥1 week sometime before baseline, number of times;  ≥5 vs. 0-4 (ref) | 63/278 vs. 215/278 | 1.51 (0.80-2.86) | .205 |
| Max length of earlier smoke-free period, number of months | md=3, Q_1_=1, Q_3_=12, N=216 | 1.01 (0.99-1.02) | .282 |
| Stages-of-change at baseline; preparation/action vs. precontemplation/contemplation (ref) | 135/278 vs. 143/278 | 1.44 (0.82-2.53) | .208 |
| Snus use the week before baseline; yes vs. no (ref) | 19/278 vs. 259/278 | 2.70 (1.04-7.03) | .042 |
| NRT use the week before baseline; yes vs. no (ref) | 22/278 vs. 256/278 | 0.32 (0.07-1.41) | .131 |
| Other support at baseline; yes vs. no (ref) | 266/278 vs. 12/278 | 3.34 (0.42-26.41) | .252 |
| Passive smoking at baseline; not exposed vs. exposed (ref) | 182/278 vs. 96/278 | 1.18 (0.65-2.14) | .597 |
| Smoking-status at 12-month follow-up; - smoker (ref) - point prevalence but <6 months - 6-month continuous abstinence | 229/284  15/284 40/284 | 1.0 4.99 (1.65-15.11) 22.44 (9.88-50.99) | .004 <.001 |
| Snus use the week before 12-month follow-up; yes vs. no (ref) | 12/209 vs. 197/209 | 1.51 (0.44-5.23) | .516 |
| NRT use the week before 12-month follow-up; yes vs. no (ref) | 34/210 vs. 176/210 | 1.05 (0.46-2.41) | .912 |
| Other support at 12-month follow-up; yes vs. no (ref) | 176/210 vs. 34/210 | 4.22 (1.23-14.41) | .022 |
| Passive smoking at 12-month follow-up; not exposed vs. exposed (ref) | 153/207 vs. 54/207 | 1.16 (0.56-2.37) | .695 |
| Compliance at 12-month follow-up; high vs. medium, low or no (ref) | 60/210 vs. 150/210 | 1.36 (0.70-2.65) | .370 |
| Snus use the week before long-term follow up; yes vs. no (ref) | 17/218 vs. 201/218 | 1.69 (0.60-4.81) | .324 |
| Drug^†^ use the week before long-term follow-up; yes vs. no (ref) | 52/238 vs. 186/238 | 0.93 (0.46-1.89) | .845 |
| NRT**^‡^** use between baseline and long-term follow-up; - none (ref) - < 5 weeks - ≥ 5 weeks | 105/225 67/225 53/225 | 1.0 0.40 (0.18-0.88) 1.08 (0.53-2.19) | .023 .837 |
| Zyban® use between baseline and long-term follow-up; - none (ref) - < 7 weeks - ≥ 7 weeks | 194/225 21/225 10/225 | 1.0 0.27 (0.06-1.18) 0.63 (0.13-3.07) | .082 .569 |
| Champix® use between baseline and long-term follow-up; - none (ref) - < 12 weeks - ≥ 12 weeks | 195/225 22/225 8/225 | 1.0 0.58 (0.19-1.79) 0.37 (0.05-3.10) | .344 .362 |
| Other support at long-term follow-up; yes vs. no (ref) | 163/236 vs. 73/236 | 2.12 (1.05-4.29) | .036 |

*n=number in category, N=total number in analysis ^†^Including NRT, Zyban®, and Champix®.
**^‡^**Max number of weeks for any preparation.
