# Supplementary material for: Long-term follow-up of a high- and a low-intensity smoking cessation intervention in a dental setting– a randomized trial
Source: BMC Public Health. 2013 Jun 19;13:592. doi: 10.1186/1471-2458-13-592 (PMC3693879; doi:10.1186/1471-2458-13-592)
Supplement: Additional file 6: Table S5 — Univariable logistic regression analyses for sustained abstinence. [file 1471-2458-13-592-S6.doc]

Additional Table 5. Univariable logistic regression analyses for sustained abstinence

| **Variable** | **n/N*** | **OR (95% CI for OR)** | **p-value** |
| --- | --- | --- | --- |
| Program; HIT vs. LIT (ref) | 141/284 vs. 143/284 | 2.66 (1.07-6.64) | .036 |
| Gender; men vs. women (ref) | 58/284 vs. 226/284 | 1.03 (0.37-2.88) | .958 |
| Age at baseline | md=49, Q_1_=42, Q_3_=56, N=278 | 1.01 (0.97-1.05) | .804 |
| Education; - 0-9 years (ref) - 10-12 years - ≥ 13 years | 61/278 115/278 102/278 | 1.0 1.21 (0.36-4.10) 1.72 (0.52-5.67) | .760 .371 |
| Number of years smoked before baseline | md=30, Q_1_=21, Q_3_=35, N=276 | 0.98 (0.94-1.01) | .221 |
| Number of cigarettes at baseline | md=105, Q_1_=70, Q_3_=140, N=278 | 0.99 (0.98-1.00) | .056 |
| Smokefree ≥1 week sometime before baseline; yes vs. no (ref) | 217/278 vs. 61/278 | 1.08 (0.38-3.01) | .891 |
| Smokefree ≥1 week sometime before baseline, number of times;  ≥5 vs. 0-4 (ref) | 63/278 vs. 215/278 | 0.66 (0.22-2.01) | .466 |
| Max length of earlier smoke-free period, number of months | md=3, Q_1_=1, Q_3_=12, N=216 | 1.01 (1.00-1.03) | .135 |
| Stages-of-change at baseline; preparation/action vs. precontemplation/contemplation (ref) | 135/278 vs. 143/278 | 1.86 (0.79-4.41) | .158 |
| Snus use the week before baseline; yes vs. no (ref) | 19/278 vs. 259/278 | 1.27 (0.28-5.85) | .761 |
| NRT use the week before baseline; yes vs. no (ref) | 22/278 vs. 256/278 | 0.38 (0.11-1.33) | .129 |
| Other support at baseline; yes vs. no (ref) | 266/278 vs. 12/278 | 1.602E8 (0.000-)^§^ | .999^§^ |
| Passive smoking at baseline; not exposed vs. exposed (ref) | 182/278 vs. 96/278 | 2.12 (0.77-5.87) | .148 |
| Smoking-status at 12-month follow-up; - smoker (ref) - point prevalence but <6 months - 6-month continuous abstinence | 229/284 15/284 40/284 | NA|| | NA|| |
| Snus use the week before 12-month follow-up; yes vs. no (ref) | 12/209 vs. 197/209 | 1.59 (0.33-7.74) | .565 |
| NRT use the week before 12-month follow-up; yes vs. no (ref) | 34/210 vs. 176/210 | 0.20 (0.03-1.55) | .123 |
| Other support at 12-month follow-up; yes vs. no (ref) | 176/210 vs. 34/210 | 4.96 (0.65-38.04) | .123 |
| Passive smoking at 12-month follow-up; not exposed vs. exposed (ref) | 153/207 vs. 54/207 | 1.39 (0.49-3.92) | .534 |
| Compliance at 12-month follow-up; high vs. medium, low or no (ref) | 60/210 vs. 150/210 | 1.94 (0.81-4.66) | .136 |
| Snus use the week before long-term follow up; yes vs. no (ref) | 17/218 vs. 201/218 | 2.05 (0.54-7.79) | .291 |
| Drug^†^ use the week before long-term follow-up; yes vs. no (ref) | 52/238 vs. 186/238 | 0.14 (0.02-1.06) | .056 |
| NRT**^‡^** use between baseline and long-term follow-up; - none (ref) - < 5 weeks - ≥ 5 weeks | 105/225 67/225 53/225 | 1.0 0.38 (0.12-1.20) 0.36 (0.10-1.30) | .100 .120 |
| Zyban® use between baseline and long-term follow-up; - none (ref) - < 7 weeks - ≥ 7 weeks | 194/225 21/225 10/225 | 1.0 0.00**^¶^** 0.00**^¶^** | .998**^¶^** .999**^¶^** |
| Champix® use between baseline and long-term follow-up; - none (ref) - < 12 weeks - ≥ 12 weeks | 195/225 22/225 8/225 | 1.0 0.00**^¶^** 0.00**^¶^** | .998**^¶^** .999**|¶** |
| Other support at long-term follow-up; yes vs. no (ref) | 163/236 vs. 73/236 | 1.10 (0.43-2.77) | .844 |

^*^ n=number in category, N=total number in analysis ^†^ Including NRT, Zyban®, and Champix®. **^‡^** Max number of weeks for any preparation.
^§^No participant without other support at baseline had been sustained abstinent.
^||^NA= not applicable.
**^¶^** No participant with Zyban® or Champix® use between baseline and long-term follow-up had been sustained abstinent.
