# Supplementary figures and images for: Long-term follow-up of a high- and a low-intensity smoking cessation intervention in a dental setting– a randomized trial
Source: BMC Public Health. 2013 Jun 19;13:592. doi: 10.1186/1471-2458-13-592 (PMC3693879; doi:10.1186/1471-2458-13-592)

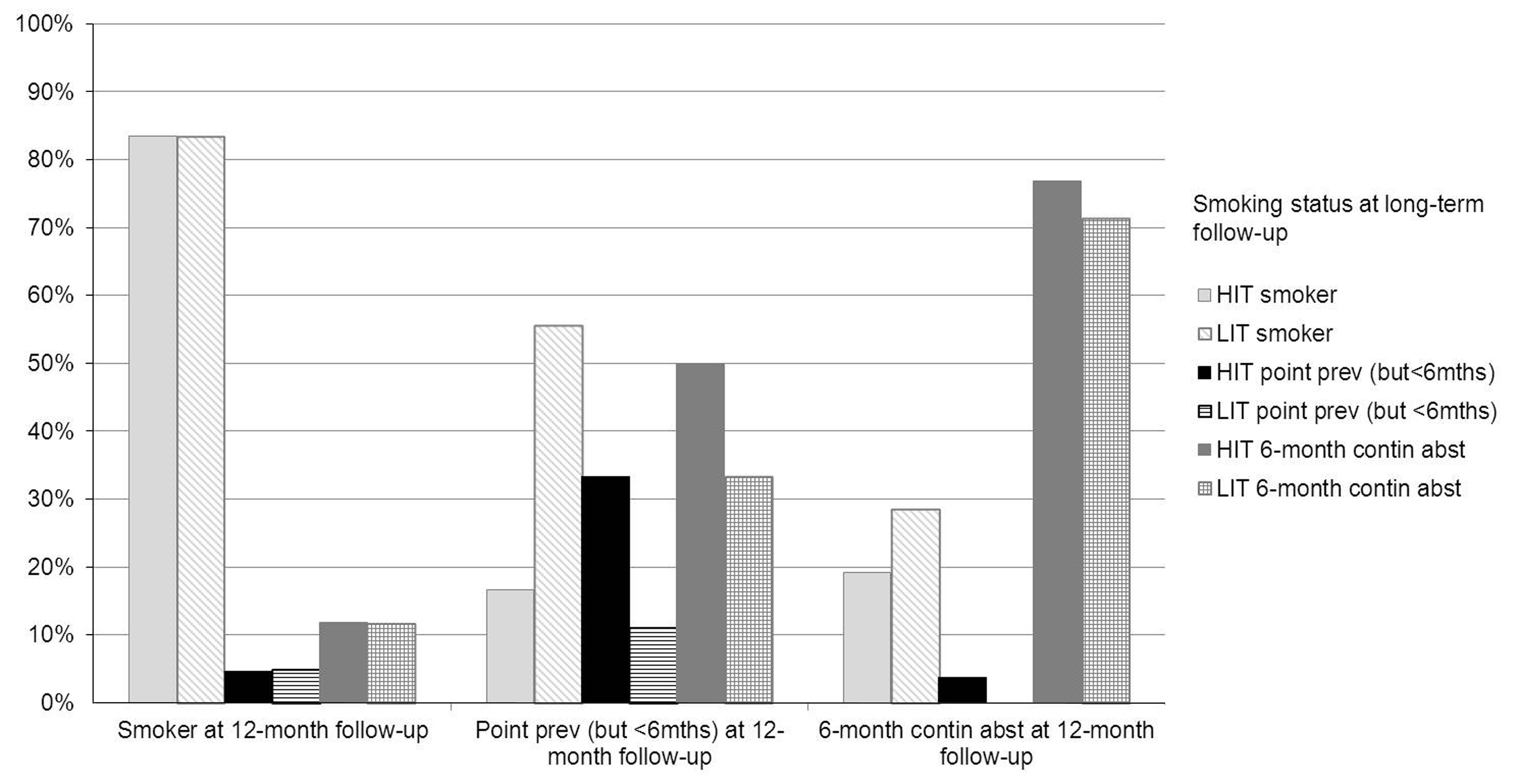

Supplement: Additional file 7: Figure S2 — Transitions between smoking statuses from 12-month to long-term (5-8ys) follow-up. [file 1471-2458-13-592-S7.tiff]
